# Gold Standard for macromolecular crystallography diffraction data

**DOI:** 10.1107/S2052252520008672

**Published:** 2020-07-10

**Authors:** Herbert J. Bernstein, Andreas Förster, Asmit Bhowmick, Aaron S. Brewster, Sandor Brockhauser, Luca Gelisio, David R. Hall, Filip Leonarski, Valerio Mariani, Gianluca Santoni, Clemens Vonrhein, Graeme Winter

**Affiliations:** a Ronin Institute for Independent Scholarship, c/o NSLS II, Brookhaven National Laboratory, Upton, New York, USA; b DECTRIS Ltd, Täfernweg 1, 5405 Baden-Dättwil, Switzerland; c Lawrence Berkeley National Laboratory, 1 Cyclotron Road, Berkeley, CA 94720, USA; d European XFEL GmbH, Holzkoppel 4, 22869 Schenefeld, Germany; e Biological Research Centre Szeged (BRC), Temesvári krt. 62, 6726 Szeged, Hungary; f University of Szeged, Arpad ter 2, 6720 Szeged, Hungary; g Center for Free-Electron Laser Science, Notkestrasse 85, 22607 Hamburg, Germany; h Diamond Light Source Ltd, Harwell Science and Innovation Campus, Didcot OX11 0DE, United Kingdom; i Swiss Light Source, Paul Scherrer Institut, Forschungsstrasse 111, 5232 Villigen PSI, Switzerland; j Structural Biology Group, European Synchrotron Radiation Facility, 71 Avenue des Martyrs, 38000 Grenoble, France; k Global Phasing Ltd, Sheraton House, Castle Park, Cambridge CB3 0AX, United Kingdom

**Keywords:** structural biology, serial crystallography, macromolecular diffraction data format, synchrotrons, XFELs, NeXus, HDF5, NXmx, CBF, imgCIF

## Abstract

Macromolecular crystallography (MX) is the dominant means of determining the three-dimensional structures of biological macromolecules. Over the last few decades, most MX data have been collected at synchrotron beamlines using a large number of different detectors produced by various manufacturers and taking advantage of various protocols and goniometries. These data came in their own formats: sometimes proprietary, sometimes open. The associated metadata rarely reached the degree of completeness required for data management according to Findability, Accessibility, Interoperability and Reusability (FAIR) principles. Efforts to reuse old data by other investigators or even by the original investigators some time later were often frustrated. In the culmination of an effort dating back more than two decades, a large portion of the research community concerned with high data-rate macromolecular crystallography (HDRMX) has now agreed to an updated specification of data and metadata for diffraction images produced at synchrotron light sources and X-ray free-electron lasers (XFELs). This ‘Gold Standard’ will facilitate the processing of data sets independent of the facility at which they were collected and enable data archiving according to FAIR principles, with a particular focus on interoperability and reusability. This agreed standard builds on the NeXus/HDF5 NXmx application definition and the International Union of Crystallo­graphy (IUCr) imgCIF/CBF dictionary, and it is compatible with major data-processing programs and pipelines. Just as with the IUCr CBF/imgCIF standard from which it arose and to which it is tied, the NeXus/HDF5 NXmx Gold Standard application definition is intended to be applicable to all detectors used for crystallography, and all hardware and software developers in the field are encouraged to adopt and contribute to the standard.

## Abstract 2

A large portion of the research community concerned with high data-rate macromolecular crystallography has agreed to an updated specification of data and metadata for diffraction images to be produced at light sources to facilitate the processing of data sets and to enable data archiving according to FAIR principles. Here, the resulting standard is presented.

## 1. Introduction   

Macromolecular crystallographic data have been captured in different ways over the past several decades:

The principal method used today for single crystal X-ray data collection is the Arndt-Wonacott screenless rotation method formalized in the late 1970s … [T]he technique of rotating a crystal around a single goniostat axis, illuminating it with monochromatic radiation, and collecting the data on a flat detector is identical. Indeed, this would not have been very surprising to the pioneers of X-ray crystallography early in the twentieth century, since the elements of this method were available in the early days of the science.

(Powell, 2019 ▸; Arndt & Wonacott, 1977 ▸), and

In the 1950s and 1960s, macromolecular crystallographic (MX) data were collected either by precession methods onto film or by single counter diffractometry. … It was clear that users would benefit from the development of a method that would provide the efficiency of film and the accuracy and automaticity of diffractometry. The "best of both worlds" would thus be a method of electronic detection that combined the advantage of both … techniques.

(Howard, 1996 ▸). By the mid-1990s such area detectors had become well established in MX, but there was a lack of agreement on a common format for the data and supporting metadata.

In 1995, Andrew Hammersley proposed a ‘Crystallographic Binary Format’ which, after considerable discussion and revision, was adopted by the IUCr in 2005 (Bernstein, 2005 ▸; Bernstein & Hammersley, 2005 ▸; Ellis & Bernstein, 2005 ▸). The resulting ‘imgCIF/CBF’ format, metadata and supporting software was adopted by Dectris for the then-new PILATUS detector in 2007 (Powell *et al.*, 2007 ▸). In subsequent years it became clear that changes would be needed to this format to support higher data rates and institutional policies (Bernstein, 2010 ▸). For the Dectris EIGER detectors, CBF was integrated with the Hierarchical Data Format (HDF5) and became the new NeXus/HDF5 NXmx format (Donath *et al.*, 2013 ▸; Könnecke *et al.*, 2015 ▸; Hester, 2016 ▸; Bernstein, 2017 ▸).

The development of electronic area detectors in macromolecular crystallography over the past few decades has meant that the vast majority of end users are unaware that there ever were alternative measurement technologies. The habit of separating the actual raw data from the necessary metadata, which was implicit when working with data on film, is now pointless and is a significant hindrance to the efficient analysis and sharing of data.

The concepts necessary to sharing data effectively, Findability, Accessibility, Interoperability and Reusability (FAIR), have long been recognized and were formalized as ‘The FAIR Guiding Principles for scientific data management and stewardship’ (Wilkinson *et al.*, 2016 ▸), and are now widely accepted. Both CBF for the PILATUS and NXmx for the EIGER have worked well within the context of data collection at specific beamlines at various facilities but, with the passage of time, variations in the choices of mandatory metadata have created difficulties in processing data collected at a given facility with software in use for data collected at other institutions, and this has required software developers to accommodate significant variations in data formats to process such data. This has been an ongoing and increasing problem since 2007, especially with respect to interoperability and reusability. This problem has been recognized by a large portion of the research community concerned with high data-rate macromolecular crystallo­graphy (HDRMX).

As noted by Kroon-Batenburg & Helliwell (2014 ▸), there have been many different approaches to defining the necessary metadata for processing crystallographic data, ranging from minimal ‘miniCBFs’ which can document many simple single-axis data sets to very complete and complex ‘full CBFs’ that document all aspects of the data collection including all settings of all positioning axes and all characteristics of the beam. The ‘full CBF’ (Bernstein & Hammersley, 2005 ▸) is an IUCr Crystallographic Information File (CIF) format file (Hall *et al.*, 1991 ▸) with the ability to support binary image files and is an extension to the Protein Data Bank mmCIF format (Westbrook & Fitzgerald, 2003 ▸). CIF, in turn, is an IUCr-authorized STAR (Self-Defining Text Archive and Retrieval) format for crystallography, and the Crystallographic Information Framework (also abbreviated CIF) is a system of exchange protocols based on data dictionaries and relational databases which is widely used in the field of crystallography. mmCIF with PDBx extensions became a master format for the PDB in 2013 and is now the only permitted format for PDB deposition (Adams *et al.*, 2019 ▸). For many years there had been resistance to recording more metadata than seemed to be needed at the time of initial data collection. Attitudes have changed as smaller, more intense beams, large numbers of small samples, much higher data rates and multimodal experiments have made the delays caused by special cases and the need to manually search for extra metadata from multiple sources unacceptable. Even such excellent ideas as including images or blueprints showing experimental setups, as in Fig. 1 in Kroon-Batenburg & Helliwell (2014 ▸), are no longer sufficient at current data rates. Parsable specifications of everything that might be needed in processing the data is now the best practice.

After two decades of effort, agreement has been reached on an updated specification of data and metadata for diffraction images to be produced at light sources:

(i) to facilitate the processing of data sets by tools available to users at a wide range of institutions, including at their home institutions as well as at light sources other than those at which they were collected, and
(ii) to ensure that software and algorithms developed in the future can be used to extract additional and new information from the raw archived data with a complete experimental description (Kroon-Batenburg *et al.*, 2017 ▸).

We call this new specification the ‘Gold Standard’ (Bernstein *et al.*, 2020 ▸). The agreed specification builds on the NeXus/HDF5 NXmx application definition (in the NeXus User Manual and Reference Documentation; http://download.nexusformat.org/doc/html/classes/applications/NXmx.html#nxmx) and the IUCr CIF imgCIF/CBF dictionary used for the ‘full CBF’ (on the IUCr Image CIF dictionary web page https://www.iucr.org/resources/cif/dictionaries/cif_img). Many of the fields of the Gold Standard are explicitly ‘required’ in all valid data sets. In order to maximize the range of use cases, other fields that are only ‘recommended’ or ‘optional’ are also specified. The specification in this paper is just given in NeXus/HDF5, but translation back to ‘full CBF’ is feasible when needed to run with older software not yet adapted to NeXus/HDF5. Even when a Gold Standard data set is written as a NeXus/HDF5 file, any or all of the CIF data needed for eventual PDB deposition may be added into the NeXus/HDF5 file using the NeXus NXpdb base class when it is available or has been calculated.

This standard is focused on raw diffraction images rather than the structure factors, since in modern MX data collection, diffraction images are the primary raw data and structure factors are derived data. Structure factors are very important, and even if they are derived data they should of course be recorded, not least because since 2008 they have been mandatory for PDB depositions using the appropriate mmCIF definitions (Jiang *et al.*, 1999 ▸). If structure factors are available, they should be added to Gold Standard files for storage, archiving and deposition. In mmCIF the REFLN category is used. In NeXus/HDF5 the NXreflections category is used.

## 2. MX and its history of sharing, openness and standards   

There is a natural tension between the desire for a scientist to work on their own data and the value to the field as a whole in sharing as much data as possible. Macromolecular crystallo­graphy has been sharing data on atomic coordinates in standardized formats since the establishment of the Protein Data Bank in 1971 (Bernstein *et al.*, 1977 ▸). For macromolecules, the PDB coordinate format became the *de facto* standard for MX. Starting in 1990, the small-molecule crystallography community began a rapid transition to standardized formats for coordinate data using the Crystallographic Information File (CIF) format (Hall *et al.*, 1991 ▸). The MX community began a discussion of a macromolecular CIF (mmCIF) for coordinate data in 1993 (Fitzgerald *et al.*, 1993 ▸). Diffraction-image formats were still very varied, however. The deposition of structure factors in the PDB was permitted from the beginning. By 1995, one quarter of PDB depositions were made with structure factors in a variety of formats favored by various software packages. By 1996, the fraction of depositions with structure factors had risen to more than half and the use of an mmCIF-based standard format for structure factors was agreed. As noted in Section 1, at the same time the MX community began serious consideration of imgCIF/CBF as a standardized open format for diffraction images.

### 2.1. A history of incomplete and incompatible metadata   

The process of adoption of a standardized open diffraction-image format has been slow. One of the most difficult-to-surmount potential barriers to adoption of a common format has been a lack of agreement about which metadata should always be incorporated with diffraction-image data. For some experiments and processing programs only the image itself is needed; all other data and metadata, such as wavelength, detector distance, rotation angles *etc.* are provided separately in ‘INP’ or ‘site’ files, or in proprietary image headers. When the PILATUS CBF image format was adopted in 2007 it was specified with complete metadata, but shortly after that the so-called ‘miniCBF’ format with much more limited metadata was adopted and has been widely used (Dectris, 2013 ▸). Because the limited list of metadata in one miniCBF collected to the standards of one facility may not be sufficient to meet the processing demands of software tuned to inconsistent lists of metadata produced at other facilities, a large number of undocumented variants of miniCBF format with idiosyncratic and inconsistent metadata have been used, necessitating searches through laboratory notebooks and other records to resolve ambiguities, as well as site-specific patches to software.

Software developers have had to code facility-specific patches and, as users have become more mobile and have needed to work at multiple facilities, various light sources have had to find solutions to these problems. Even though data collection and processing were already becoming much faster, the common mindset was that data collection took significant beam time and computer time, and that lack of completeness and consistency in metadata was considered a relatively minor issue at many facilities. The occasional nuisance of searching for missing metadata was accepted as a reasonable cost to pay for the convenience of a short, simple list of required metadata. In addition, software developers were very obliging and did an excellent job of adapting their code to the large variety of metadata in use by making many options available in their command lines, INP files and site files, hiding the cost of translating actual collection metadata in multiple places to software-specific metadata.

### 2.2. Transformation of MX using hybrid photon-counting (HPC) detectors with high data rates and volumes   

In 2007 the first PILATUS detectors strained then-available computers and networks with 10–12.5 six-megapixel frames per second, producing approximately 2–2.4 gigabits [0.25–0.3 gigabytes (GB)] of data per second before compression (Schulze-Briese, 2007 ▸; Kraft *et al.*, 2009 ▸). EIGER detectors are now capable of 133 18-megapixel frames per second, and the latest EIGER2 XE can generate 400–550 frames per second, producing up to 160 gigabits (20 GB) of data per second before compression (Dectris, 2020 ▸).

Except for very sparsely populated images, the available lossless compressions improve the data rates by at best one order of magnitude.

At the European XFEL, which generates 27 000 X-ray pulses per second (Decking *et al.*, 2019 ▸), AGIPD, LPD and DSSC detectors are collecting one megapixel images with 0.22 µs time separation between them at frame rates of 3520, 5110 and 8000 frames per second, respectively (Hauf *et al.*, 2019 ▸). This results in up to 256 gigabits of data per second (32 GB/s) before compression.

Over the last decade, new beamlines with smaller, more intense beams have resulted in data-acquisition times that are two or more orders of magnitude shorter, with no sign of this progress slowing down. This has been coupled with massive improvements in sample-handling automation so that overall throughput is increasing, thereby placing an ever-increasing emphasis on automated processing, hence the need for reliable and trustworthy metadata. We are long past the point where incomplete or inconsistent metadata can be tolerated.

Over the last 13 years, since the introduction of the PILATUS detector using CBF and miniCBF, the landscape has changed, with the gain in popularity of artificial intelligence and machine learning, the internet of things and big data paradigms in the IT industry. These have come with new hardware techniques, using for example graphical processing units (GPUs) for highly parallel computations and field-programmable gate arrays (FPGAs) for real-time processing. It will be much easier to adapt our processing pipelines to these new technologies if we standardize the content of our metadata and ensure that all of the essential elements needed for processing are readily accessible. If the standards are respected then it is likely that developers will have time to focus on more important technical challenges.

### 2.3. Hardware, software, automation and the need for standards   

As presented above, detector data rates have increased by roughly 100-fold over the last 13 years, while the data throughput of CPU-based computing systems has improved much more slowly (Thompson, 2017 ▸; Thompson & Spanuth, 2018 ▸; Patterson, 2018 ▸; Hennessy & Patterson, 2019 ▸). State-of-the art server systems are already saturating with data. Currently, the best-performing CPUs are reaching 1 Tbit/s (125 GB/s) memory throughput (per socket). Peripheral interfaces can be connected at 500 Gbit/s (62.5 GB/s) with PCI Express 4 and at 400 Gbit/s (50 GB/s) with OpenCAPI. Higher speeds are only available in GPU-specific interfaces, which reach 1.2 Tbit/s (150 GB/s; Roberts *et al.*, 2018 ▸; Vergara Larrea *et al.*, 2019 ▸).

The data rate of the fastest commercially available implementation of an Ethernet standard is 400 Gbit/s (50 GB/s). While memory throughput can be increased by an order of magnitude with on-chip high-bandwidth memory, peripheral interfaces for input/output will at best double in the next 2–3 years according to published industry plans (Song *et al.*, 2019 ▸).

As a consequence, the major bottleneck in diffraction-image processing is the movement of data. State-of-the-art server systems are already saturating with data (Leonarski *et al.*, 2020 ▸). All unnecessary transfers or conversions of image data need to be avoided. In addition, most of the software that is in current use was designed in the context of processors supporting very little parallelism, even though the increasing demand for automation in response to higher detector speeds and more intense beams can only be satisfied by higher levels of parallelism. Unfortunately, the necessary algorithmic changes are challenging to address. We are in the peculiar position where the easiest step to take to meet the need for higher performance is to adopt uniform standards for data and metadata so that as few conversions and data motions as possible are needed.

New methods, such as for example serial crystallography at synchrotrons and XFELs, also result in new software development. Broad adoption of the Gold Standard will help to ensure that data and metadata are consistently read by all the available software.

### 2.4. Data archiving (FAIR)   

While the immediate benefit for uniform MX standards is in achieving the best performance, uniform MX data and metadata standards also make it easier to prepare data sets for archiving (Helliwell, 2019 ▸). This then facilitates searches and reuse of the raw data, both for better processing with future improved methods and in the use of known crystallographic structures for molecular replacement for the determination of new crystallographic structures or as higher resolution components of cryoEM images of large molecular machines.

## 3. History of HDRMX meetings and Gold Standard development   

Since 2016, beamline scientists, controls scientists, data-acquisition scientists, data-analysis scientists and others with an interest in high data-rate macromolecular crystallography have been meeting occasionally to explore ways to improve the processing of crystallographic data from the newest generations of detectors. Documentation of these discussions can be found at http://hdrmx.medsbio.org. There was discussion of the need for appropriate minimal metadata at all of the HDRMX meetings, but agreement on trying to formalize a Gold Standard began at the HDRMX meeting at ACA 2018 in Toronto, Canada on 22 July 2018, continued with further discussion at the HDRMX Satellite to AsCA 2018/Crystal 32 in Auckland, New Zealand on 6–7 December 2018, at the HDRMX meeting at ACA 2019, Covington, Kentucky, USA on 21 July 2019 and at the HDRMX meeting at ECM32, Vienna, Austria on 20 August 2019, and achieved final agreement on the Gold Standard at the HDRMX meeting at Diamond Light Source, Chilton, United Kingdom on 6–7 November 2019.

## 4. Description of the Gold Standard and compliance with software   

Whether we are dealing with CBF files or NeXus/HDF5 files, the information in a Gold Standard data set is the same: one or more diffraction-image data arrays of pixels along with sufficient metadata to allow software to determine exactly where in the laboratory coordinate system each pixel was located and when the intensity recorded in that pixel was recorded, so that the software can locate spots, index them and integrate them. For example, the conversion of pixel positions relative to the detector to reciprocal-space positions requires knowledge of the pixel size, the detector distance, the detector orientation, the wavelength and the beam center. In the past some of the metadata needed for this process might have been recorded in the same set of files as the image-data arrays and some of the necessary metadata might have been recorded elsewhere, for example in a laboratory notebook or in some separate electronic laboratory notebook. In a Gold Standard data set, the necessary data and metadata for processing a reasonable range of use cases is recorded in the data set. This allows the data set to be moved freely to other filesystems in other facilities and still be processed, without the need to return to the original facility to recover information that had been left behind. Although the data set will normally consist of multiple files, these files should be packaged together in an appropriate container, for example a single folder in the file system at the collecting facility or under a single DOI in a data-set repository.

The specification of which metadata need be retained with the data depends on the experiment being performed and the software that will be used for processing, *i.e.* the ‘use case’. The Gold Standard being discussed here is intended to be adequate for single-axis rotation experiments at synchrotrons and stills collected at XFELs and synchrotrons, and to work properly with the data-reduction programs *DIALS* (Waterman *et al.*, 2013 ▸; Winter *et al.*, 2018 ▸), *XDS* (Kabsch, 2010*a* ▸,*b* ▸), *MOSFLM* (Battye *et al.*, 2011 ▸), *HKL*-2000 (Otwinowski & Minor, 1997 ▸), *xia*2 (Winter, 2010 ▸) and *autoPROC* (Vonrhein *et al.*, 2011 ▸), as well as future versions of *OnDA* (Mariani *et al.*, 2016 ▸). The more complex the design of the experiment and the more varied the non-default choices permitted by the software, the more different metadata may be required to ensure correct processing at a wide range of facilities. The Gold Standard is the minimum set of metadata upon which we have agreed.

To date, the applicability of the Gold Standard has been demonstrated both for single-axis rotation data at a synchrotron (https://doi.org/10.5281/zenodo.3484187) and for serial crystallography data at an XFEL (https://doi.org/10.5281/zenodo.3352357).

The former is a ‘Small example Eiger2 X 16M data set from Diamond Light Source I04 revised for HDRMX Gold Standard Discussion’ collected by Graeme Winter and revised by Graeme Winter, Aaron Brewster and Herbert J. Bernstein to conform to the Gold Standard. On Zenodo this is described as a

Revised useful small (488 frame) Eiger data set recorded during routine testing, useful for software testing as it is small. [The recorded data are] from a thaumatin crystal by Graeme Winter, The original dataset is https://doi.org/10.5281/zenodo.3385862 which contains two Eiger2 X datasets, Therm_6_1 and Therm_6_2, each with a data file and two versions each of the metadata – a "…_master.h5" file and a "….nxs" file. The former are the usual Eiger metadata files using exposed external links to connect the metadata to the data, and the latter are HDF5-1.10 VDS (virtual dataset) files. This revision has the same data as the original Therm_6_2 data, but now includes with the "master.h5" file a "…_master_rev.h5" file and with the ".nxs" file a "…_rev.nxs" VDS file.

The latter is a ‘68 image lysozyme dataset recorded on the Jungfrau 16M detector at SwissFEL and formatted as a NeXus file’ by Aaron Brewster, Karol Nass, Dmitry Ozerov and Filip Leonarski. Minor changes for Gold Standard compliance were made by Herbert J. Bernstein. The Zenodo reference at https://doi.org/10.5281/zenodo.3352357, in addition to the data set described here, contains instructions on how to generate the NeXus master files using a Python script. The data set was collected with a JUNGFRAU 16M hybrid pixel charge-integrating detector (Redford *et al.*, 2018 ▸) during SwissMX fixed-target instrument commissioning at the SwissFEL Bernina endstation (Ingold *et al.*, 2019 ▸). The detector is composed of 32 independent modules, each of roughly 500 000 pixels, arranged in a non-square geometry and mounted in a single metal frame. To simplify refinement of the module positions, we grouped the detector modules into a hierarchy of four quadrants with eight modules per quadrant. Each module consists of eight panels in a 4 × 2 arrangement.

In order to represent this arrangement in NeXus, we used a series of NXtransformations nodes linked by the depends_on attribute to represent (i) the position of the detector as a whole relative to the crystal, (ii) the location of the quadrants relative to the detector center, (iii) the location of the eight modules in each quadrant relative to their quadrant center and (iv) the location of each of the eight panels in a module relative to their module center. We included pixel offsets for each panel into the raw data following the NXmx specification.

**Figure 1:**
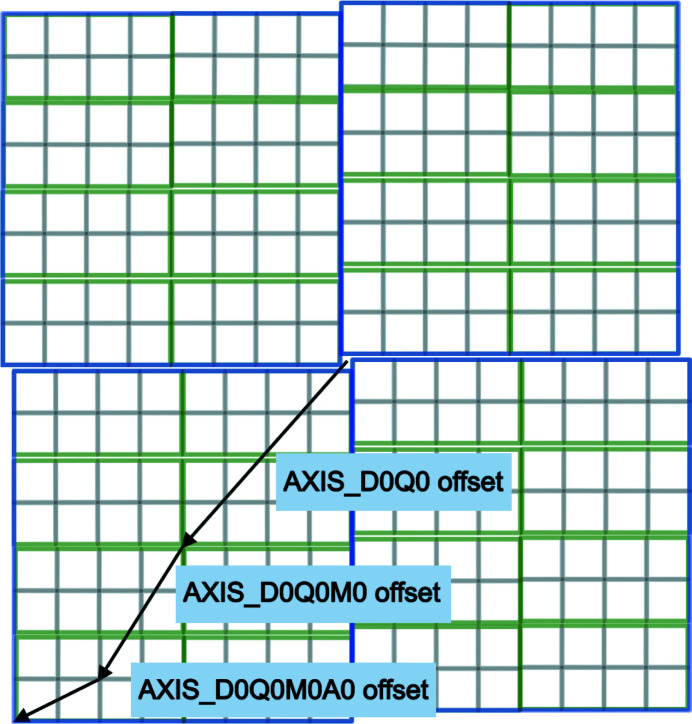
Schematic of the JF16M detector viewed from the source side, showing the hierarchical arrangement of panels. The quadrants are outlined in blue, the modules in green and the ASICs in gray. The offset components of the NeXus transformations are shown as arrows for the quadrant zero, sensor zero and ASIC zero. Note that the arrows point in the directions of the offsets, which are in the *X*–*Y* plane, not in the directions of the axes themselves, which are in the *Z* direction.

The JUNGFRAU 16M (JF16M) represents a complex detector geometry and so an illustrative example is helpful. The following example shows the depends_on chain for the JF16M SwissFEL data set for the zeroth (first) panel. The chain starts with the NXdetector_module group at /entry/instrument/ELE_D0/ARRAY_D0Q0M0A0. The group is named after its hierarchy, referring to the fact that it is ASIC (application specific integrated chip) zero, of module zero, of quadrant zero, of detector zero. It has two fields that define the orientation of the panel by specifying the fast and slow readout directions for the raw pixel data:
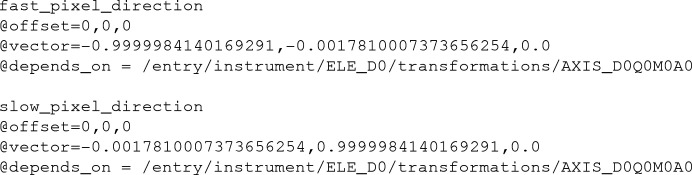


Both the fast and slow pixel directions depend on the AXIS_D0Q0M0A0 field of the NXtransformations group at /entry/instrument/ELE_D0/transformations, and this field continues the dependency chain of detector groups until the final group, which is the detector rail:
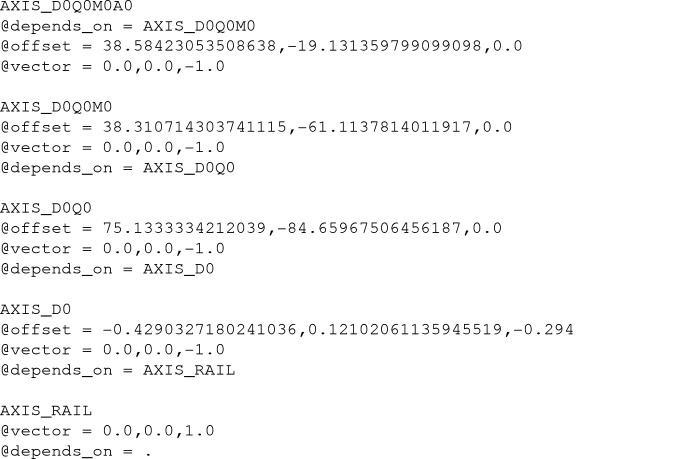


(see Fig. 1 ▸). Each of the other panels defines a similar set of transforms. We note here that this hierarchical arrangement of axes is similar to work performed previously for the CSPAD CBF format (Brewster *et al.*, 2014 ▸). Aaron Brewster and Asmit Bhowmick at LBL, together with Yury Kirienko, Fabio Dall’Antonia and Sandor Brockhauser at EuXFEL, have also worked on similar NeXus master files for the EuXFEL AGIPD detector (to be discussed in a future publication).

### 4.1. Identifying the provenance of the data   

While each data set should contain all of the data and metadata necessary for processing, it also should clearly identify where and when it was collected by specifying the scientific instrument or beamline and the facility at which it was collected and the times of collection. In the NXmx Gold Standard, the full name of the scientific instrument or beamline is carried in the /(entry):NXentry/(instrument):NXinstrument/name field and the name of the facility is carried in the /(entry):NXentry/(source):NXsource/name field. The commonly used acronyms or abbreviations of each of the names in these name fields are carried in the associated @short_name attributes. The full and precise UTC ISO 8601 (Wolf & Wicksteed, 1998 ▸) time/date of the first data point collected is carried in the (entry):NXentry/start_time field and an estimate of the likely time of collection of the last data point is carried in the /(entry):NXentry/end_time_estimated field. If/when the data collection is completed, the full and precise UTC ISO 8601 time/date of the last data point collected is carried in the /(entry):NXentry/end_time field, provided that it is accurately observed. The time zone of the beamline is carried in the /(entry):NXentry/(instrument):NXinstrument/time_zone field so local times may be recovered.

In most instances, one could process a data set with a missing ‘start_time’ or with a missing ‘end_time’ but, as a matter of accurate scientific record keeping, both should be included if possible. This will help if later in the life of the data set it is necessary to correlate information in this data set in time sequence with information in other data sets or other records. As a practical matter, if we have any data at all the ‘start_time’ is known accurately, but if the collection aborts we may not have an accurate ‘end_time’. Therefore, in the Gold Standard NXmx, ‘start_time’ and ‘end_time_estimated’ are required, since we can estimate the latter from the former plus the frame rate. We have made ‘end_time’ optional, but if it has been observed then it should be included.

Caution is needed when dealing with time zones and daylight saving time, especially when trying to correlate information from different data sets. For this reason, all times should be given without using any local time zone, *i.e.* all times should be given as UTC times using the ‘Z’ suffix. The local time zone should be given in /(entry):NXentry/(instrument):NXinstrument/time_zone to allow the recovery of local times when needed.

### 4.2. Experimental geometry   

**Figure 2:**
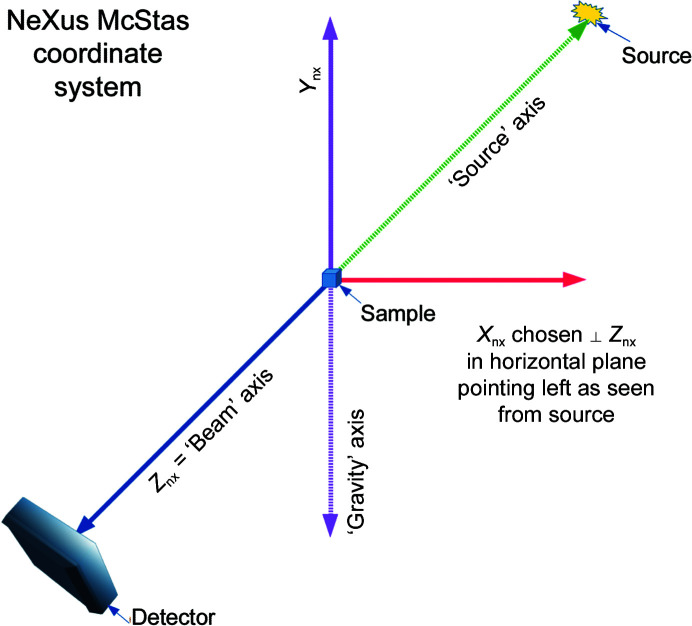
The NeXus/HDF5 files specify axes in the NeXus McStas coordinate system. The standard coordinate frame in NeXus is the McStas coordinate frame, in which the *Z* axis points in the direction of the incident beam, the *X* axis is orthogonal to the *Z* axis in the horizontal plane and pointing left as seen from the source, and the *Y* axis points upwards to form a right-handed axis system. The origin is in the sample.

**Figure 3:**
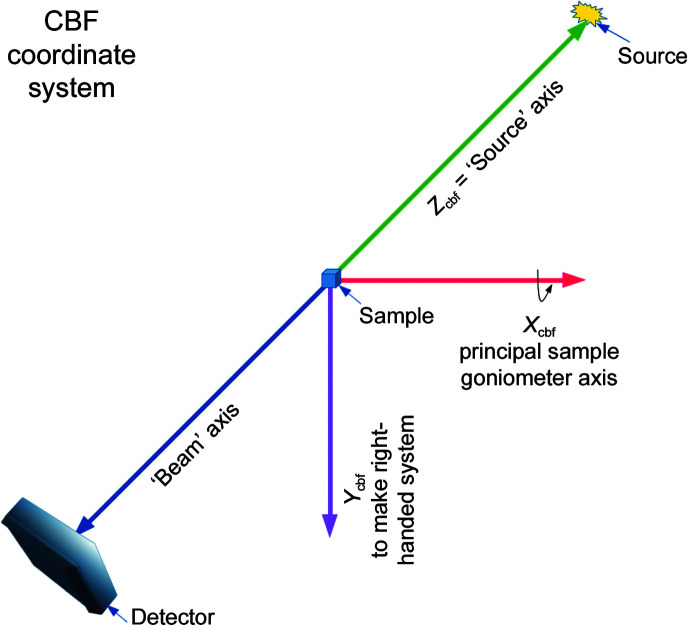
The standard coordinate frame in imgCIF/CBF aligns the *X* axis with the principal goniometer axis and chooses the *Z* axis to point from the sample into the beam, *i.e.* the ‘Source’ vector. If the beam is not orthogonal to the *X* axis, the *Z* axis is the component orthogonal to the *X* axis of the ‘Source’ (or ‘−Beam’) vector. The *Y* axis is chosen to complete a right-handed axis system. The origin is in the sample. It is important to note that the direction of the principal goniometer axis is a design choice in creating or even in configuring a crystallographic beamline. Even if we were to restrict our choices of principal goniometer axes to be horizontal, it is possible and equally valid to have CBF coordinate frames in which the *Y* axis points down, as in this figure, or to have CBF coordinate frames in which the *Y* axis points up, depending on the direction of the *X* axis.

One of the most important sets of metadata used in processing is information on where the components of the experimental setup are positioned and oriented relative to one another. We need to precisely map the events recorded in a pixel to reciprocal space, which implies a need to know or infer the sample orientation, detector position and characteristics, beam wavelength and direction at the very least. We need to know how the sample is positioned and oriented relative to the incident beam, where the detector is positioned and oriented relative to the sample, where in the plane of the detector the incident beam would have hit and where the various sensor modules of the detector are positioned relative to one another. Essentially, we need a blueprint of the experimental setup. The set of metadata used for this purpose both in CBF and in NeXus/HDF5 describes fixed or variable positioning axes in terms of directional vectors in nested lists with optional offset vectors between pairs of axes. For an experiment with both a detector and a sample goniometer, we need to provide the nested chains of axes that determine the position and orientation of the detector and of the sample. In each case we perform this backwards, starting with a specification in the description of the detector of a depends_on field specifying the axis that actually supports the detector and a specification in the description of the sample of a depends_on field specifying the axis that actually supports the sample. For each axis that is supported by another axis, we describe that axis next, until we reach a fixed point in the beamline, denoted by a ‘.’. For both CBF and NeXus, the origin of the coordinate system used is intended to be on or at the sample. If there is a sample-rotation axis, the origin is at the point on the beam where the rotation axis approaches the beam most closely (for example intersects it; Zeldin *et al.*, 2013 ▸). If there is no sample-rotation axis, the midpoint of the line segment marking the intersection of the beam with the sample is usually specified as the origin. The axes of the NeXus/HDF5 coordinate system are described in Fig. 2 ▸ and the axes of the CBF coordinate system are described in Fig. 3 ▸. In most cases the two coordinate frames are related by a 180° rotation around the vertical axis.

All axis chain definitions and axis settings necessary to process the data should be clearly and explicitly described. There are cases where the values for axis settings available at the time of data collection are only approximate. In such cases, updated or refined values may be added when later calibrations and refinements make them available. Both NeXus and CBF permit the declaration of ‘variants’ to record such cases.

The names used for particular axes are arbitrary, provided that they are used in a consistent manner, but it is good practice to use names that enhance rather than detract from understanding. In particular if ‘Beam’ is used as an axis name it should point in the direction going from the source to the sample, and if ‘Source’ is used as an axis name it should point in the direction going from the sample to the source. It is also best never to use the same axis name in two different contexts. For example, we may well have one *X* axis for the coordinate frame, another for the entire detector face and several more for each of the detector modules. Giving each use a distinct name, such as *X*_nx, *X*_detector, *X*_module_1 *etc.*, can help to avoid confusion.

In a NeXus/HDF5 NXmx file the axis chain descriptions begin with the depends_on field and NXtransformations group in each NXdetector group and in each NXsample group. In addition, the axis of the beam direction, which we call ‘Beam’, and of the downward direction of gravity, which we call ‘Gravity’, will be specified because they are needed in the coordinate system used by NeXus, which is called McStas (see Section 4.2.1).

The axes pointed to from each depends_on field should be placed in appropriate NXtransformations groups. Each axis has a dimensionless unit vector and an optional offset vector specifying the direction cosines of the axis and the offset from the previous axis in the chain to the base of the new axis.

#### 4.2.1. NeXus McStas coordinate system   

The NeXus/HDF5 files specify axes in the NeXus McStas coordinate system. It is important to note that imgCIF/CBF uses a different coordinate system. The McStas coordinate frame (Lefmann & Nielsen, 1999 ▸) is the NeXus standard coordinate frame, in which the *Z* axis points in the direction of the incident beam, ‘Beam’, going from the sample away from the source, the *X* axis is orthogonal to the *Z* axis in the horizontal plane and pointing left as seen from the source, and the *Y* axis points upwards. The origin is in the sample. It is very helpful to explicitly include the definitions of ‘Beam’ and ‘Gravity’ in an NXtransformations group to ensure that the metadata fully document the relationships. Doing this reduces the need to search out the literature on the McStas coordinate system in order to understand where these essential axes are located.

#### 4.2.2. CBF coordinate system   

The standard coordinate frame in imgCIF/CBF aligns the *X* axis with the principal gonio­meter axis and chooses the *Z* axis to point from the sample into the beam, *i.e.* be a ‘Source’ vector. If the beam is not orthogonal to the *X* axis, the *Z* axis is the component of the ‘Source’ vector orthogonal to the *X* axis. The *Y* axis is chosen to complete a right-handed axis system. The origin is in or on the sample. It is good practice to explicitly give the Beam vector and/or its negative, Source, to ensure that the metadata fully document the relationships.

### 4.3. Dealing with things that are required but not available   

The Gold Standard includes groups, fields and attributes that are ‘required’ but which may not be available until after data collection is completed or which may not ever become available. For example, a dark-field run would not have a sample, but the NXsample group is always required to form a valid NXmx entry. In such cases an appropriate null value should be used. For a group such as NXsample, a value for the field name of ‘.’ is an appropriate way to indicate that no sample has been provided. For floating-point numeric values, the special IEEE standard ‘NaN’ is a suitable null value. For example, for a collection at a facility for which the correct sensor material and thickness are not known to the experimenter at the time of data collection but are intended to be filled in later, null values should be used for these field values. For a string-valued field, such as sensor_material, ‘.’ is a good null value to use. When there is an intention to suggest that a non-null value should be provided for a ‘.’ string, the alternative null value ‘?’ can be used. For the numeric sensor_thickness, ‘NaN’ can be used when the information is not available. For fields with integer values there is no simple general null value available, but if maximum or minimum values are specified then values outside this range are suitable null values. For example, for a pixel value in a photon-counting detector, negative values can fill this role.

## 5. Summary and conclusions   

Built on the NeXus/HDF5 NXmx application definition and the International Union of Crystallography (IUCr) imgCIF/CBF dictionary, the new Gold Standard for MX diffraction data collected at synchrotrons and XFELs is compatible with major data-processing programs and pipelines, and will make it faster and easier to process MX data and will help the community to share data and metadata conforming to FAIR principles. A tree diagram of the NXmx Gold Standard and the Gold Standard NeXus NXmx application definition are provided as supporting information.

## Supplementary Material

Tree diagram of NXmx Gold Standard. DOI: 10.1107/S2052252520008672/ti5018sup1.pdf

Gold Standard NeXus NXmx application definition. DOI: 10.1107/S2052252520008672/ti5018sup2.pdf

Small example Eiger 2X 16M data set from Diamond Light Source I04 revised for HDRMX Gold Standard Discussion: https://doi.org/10.5281/zenodo.3484187

68 image lysozyme dataset recorded on the Jungfrau 16M detector at SwissFEL and formatted as a NeXus file: https://doi.org/10.5281/zenodo.3352357
