# Supplementary material for: Gold Standard for macromolecular crystallography diffraction data
Source: IUCrJ. 2020 Jul 10;7(Pt 5):784–92. doi: 10.1107/S2052252520008672 (PMC7467160; doi:10.1107/S2052252520008672)
Supplement: Supplementary file 1 [file m-07-00784-sup1.pdf]

IUCrJ

Volume 7 (2020)

Supporting information for article:

Gold Standard for macromolecular crystallography diffraction data

Herbert J. Bernstein, Andreas Förster, Asmit Bhowmick, Aaron S. Brewster, Sandor Brockhauser, Luca Gelisio, David R. Hall, Filip Leonarski, Valerio Mariani, Gianluca Santoni, Clemens Vonrhein and Graeme Winter

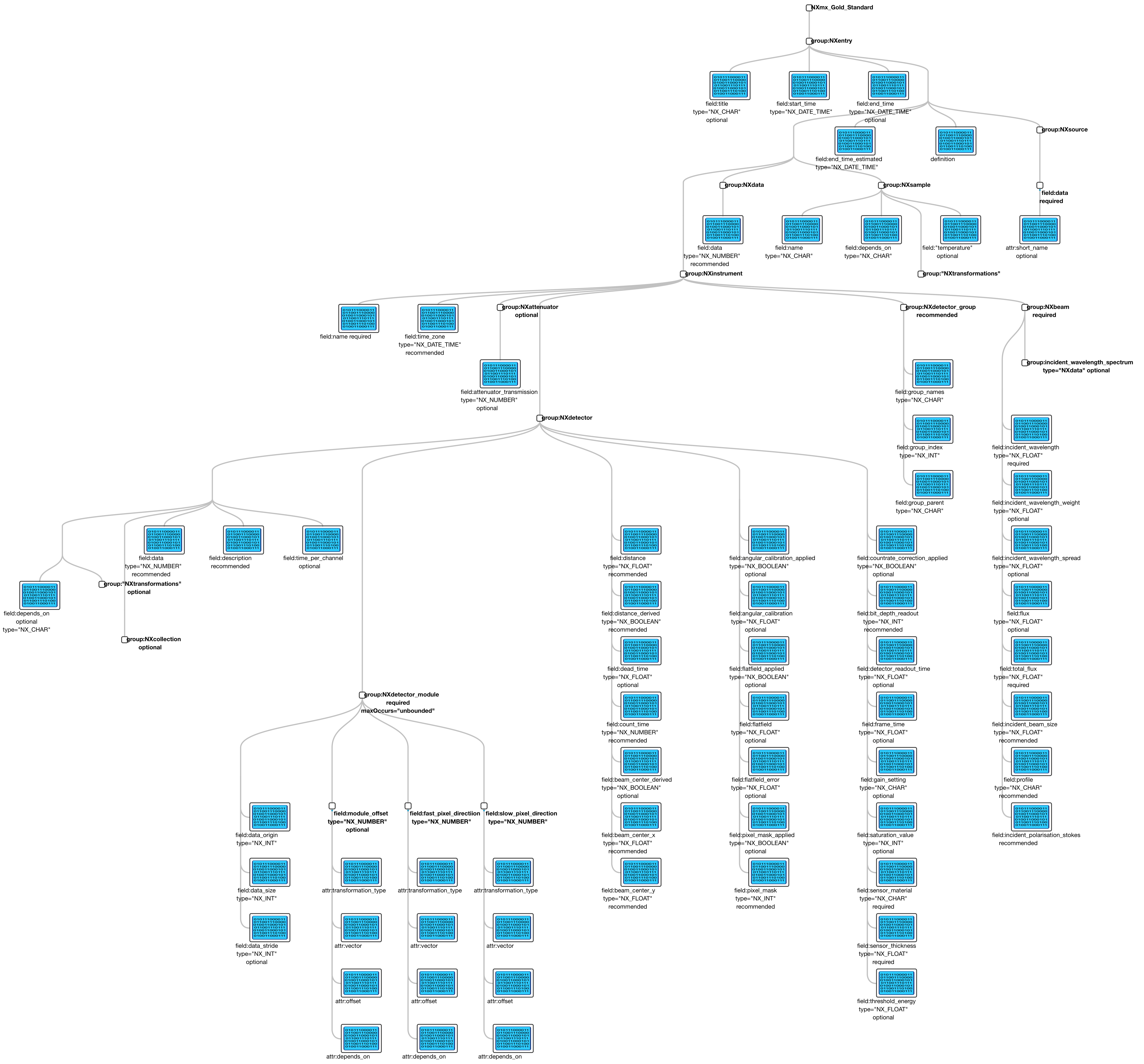
NXmx_Gold_Standard
group:NXentry
field:title
type="NX_CHAR"
optional
field:start_time
type="NX_DATE_TIME"
field:end_time
type="NX_DATE_TIME"
optional
field:end_time_estimated
type="NX_DATE_TIME"
definition
group:NXsource
field:data
required
attr:short_name
optional
group:NXdata
field:data
type="NX_NUMBER"
recommended
group:NXsample
field:name
type="NX_CHAR"
field:depends_on
type="NX_CHAR"
field:"temperature"
optional
group:"NXtransformations"
group:NXinstrument
field:name required
field:time_zone
type="NX_DATE_TIME"
recommended
group:NXattenuator
optional
field:attenuator_transmission
type="NX_NUMBER"
optional
group:NXdetector_group
recommended
field:group_names
type="NX_CHAR"
field:group_index
type="NX_INT"
field:group_parent
type="NX_CHAR"
group:NXbeam
required
group:incident_wavelength_spectrum
type="NXdata" optional
field:incident_wavelength
type="NX_FLOAT"
required
field:incident_wavelength_weight
type="NX_FLOAT"
optional
field:incident_wavelength_spread
type="NX_FLOAT"
optional
field:flux
type="NX_FLOAT"
optional
field:total_flux
type="NX_FLOAT"
required
field:incident_beam_size
type="NX_FLOAT"
recommended
field:profile
type="NX_CHAR"
recommended
field:incident_polarisation_stokes
recommended
group:NXdetector
field:data
type="NX_NUMBER"
recommended
field:description
recommended
field:time_per_channel
optional
field:depends_on
optional
type="NX_CHAR"
group:"NXtransformations"
optional
group:NXcollection
optional
field:distance
type="NX_FLOAT"
recommended
field:distance_derived
type="NX_BOOLEAN"
recommended
field:dead_time
type="NX_FLOAT"
optional
field:count_time
type="NX_NUMBER"
recommended
field:beam_center_derived
type="NX_BOOLEAN"
optional
field:beam_center_x
type="NX_FLOAT"
recommended
field:beam_center_y
type="NX_FLOAT"
recommended
field:angular_calibration_applied
type="NX_BOOLEAN"
optional
field:angular_calibration
type="NX_FLOAT"
optional
field:flatfield_applied
type="NX_BOOLEAN"
optional
field:flatfield
type="NX_FLOAT"
optional
field:flatfield_error
type="NX_FLOAT"
optional
field:pixel_mask_applied
type="NX_BOOLEAN"
optional
field:pixel_mask
type="NX_INT"
recommended
field:countrate_correction_applied
type="NX_BOOLEAN"
optional
field:bit_depth_readout
type="NX_INT"
recommended
field:detector_readout_time
type="NX_FLOAT"
optional
field:frame_time
type="NX_FLOAT"
optional
field:gain_setting
type="NX_CHAR"
optional
field:saturation_value
type="NX_INT"
optional
field:sensor_material
type="NX_CHAR"
required
field:sensor_thickness
type="NX_FLOAT"
required
field:threshold_energy
type="NX_FLOAT"
optional
group:NXdetector_module
required
maxOccurs="unbounded"
field:data_origin
type="NX_INT"
field:data_size
type="NX_INT"
field:data_stride
type="NX_INT"
optional
field:module_offset
type="NX_NUMBER"
optional
attr:transformation_type
attr:vector
attr:offset
attr:depends_on
field:fast_pixel_direction
type="NX_NUMBER"
attr:transformation_type
attr:vector
attr:offset
attr:depends_on
field:slow_pixel_direction
type="NX_NUMBER"
attr:transformation_type
attr:vector
attr:offset
attr:depends_on
