# Supplementary material for: Gold Standard for macromolecular crystallography diffraction data
Source: IUCrJ. 2020 Jul 10;7(Pt 5):784–92. doi: 10.1107/S2052252520008672 (PMC7467160; doi:10.1107/S2052252520008672)
Supplement: Supplementary file 2 [file m-07-00784-sup2.pdf]

IUCrJ

Volume 7 (2020)

Supporting information for article:

Gold Standard for macromolecular crystallography diffraction data

Herbert J. Bernstein, Andreas Förster, Asmit Bhowmick, Aaron S. Brewster, Sandor Brockhauser, Luca Gelisio, David R. Hall, Filip Leonarski, Valerio Mariani, Gianluca Santoni, Clemens Vonrhein and Graeme Winter

1. Simplified NXmx layout

A tree representation of this simplified NXmx layout is available at

http://hdrmx.medsbio.org/gold2/NXmx_Gold_Standard.jpg

```
<?xml version="1.0" encoding="UTF-8"?>
<definition name="NXmx" extends="NXobject" type="group"
    category="application"
    xmlns="http://definition.nexusformat.org/nxdl/3.1"
    xmlns:xsi="http://www.w3.org/2001/XMLSchema-instance"
    xsi:schemaLocation="http://definition.nexusformat.org/nxdl/3.1 ../nxdl.xsd" >
        <group:NXentry>
            <field:title type="NX_CHAR" optional="true" />
            <field:start_time type="NX_DATE_TIME" />
            <field:end_time type="NX_DATE_TIME" optional="true" />
            <field:end_time_estimated type="NX_DATE_TIME" />
            <field:definition />

            <group:NXdata>
                <field:data type="NX_NUMBER" recommended="true"/>
            </group:NXdata>

            <group:NXsample>
                <field:name type="NX_CHAR" />
                <field:depends_on type="NX_CHAR" />
                <group:"NXtransformations" recommended="true" />
                <field:"temperature" units="NX_TEMPERATURE" optional="true" />
            </group:NXsample>

            <group:NXinstrument>
                <field:name required />
                <field:time_zone type="NX_DATE_TIME" recommended="true" />

                <group:NXattenuator optional="true">
                    <field:attenuator_transmission type="NX_NUMBER" units="NX_UNITLESS" optional />
                </group:NXattenuator>

                <group:NXdetector_group recommended="true" >
                    <field:group_names type="NX_CHAR" />
                    <field:group_index type="NX_INT" />
                    <field:group_parent type="NX_INT" />
                </group:NXdetector_group>
                <group:NXdetector>
                    <field:depends_on optional type="NX_CHAR" />
                    <group:"NXtransformations" recommended="true" />
                    <group:NXcollection optional />

                    <field:data type="NX_NUMBER" recommended="true" />
                    <field:description recommended="true" />
                    <field:time_per_channel units="NX_TIME" optional="true" />

                    <group:NXdetector_module required maxOccurs="unbounded">
                        <field:"data_origin" type="NX_INT" />
                        <field:"data_size" type="NX_INT" />
                        <field:data_stride type="NX_INT" optional="true" />
```

```
        <field:module_offset units="NX_LENGTH" type="NX_NUMBER" optional="true" >
            <attribute:transformation_type />
            <attribute:vector type="NX_NUMBER" />
            <attribute:offset type="NX_NUMBER" />
            <attribute:depends_on />
        </field:module_offset>
        <field:fast_pixel_direction units="NX_LENGTH" type="NX_NUMBER">
            <attribute:transformation_type />
            <attribute:vector type="NX_NUMBER" />
            <attribute:offset type="NX_NUMBER" />
            <attribute:depends_on />
        </field:fast_pixel_direction>
        <field:slow_pixel_direction type="NX_NUMBER" units="NX_LENGTH">
            <attribute:transformation_type />
            <attribute:vector type="NX_NUMBER" />
            <attribute:offset type="NX_NUMBER" />
            <attribute:depends_on />
        </field:slow_pixel_direction>
    </group:NXdetector_module>

    <field:distance type="NX_FLOAT" units="NX_LENGTH" recommended="true" />
    <field:distance_derived type="NX_BOOLEAN" units="NX_LENGTH" recommended />
    <field:dead_time type="NX_FLOAT" units="NX_TIME" optional="true" />
    <field:count_time type="NX_NUMBER" units="NX_TIME" recommended="true" />
    <field:beam_center_derived type="NX_BOOLEAN" units="NX_LENGTH" optional="true" />
    <field:beam_center_x type="NX_FLOAT" units="NX_LENGTH" recommended="true" />
    <field:beam_center_y type="NX_FLOAT" units="NX_LENGTH" recommended="true" />
    <field:angular_calibration_applied type="NX_BOOLEAN" optional="true" />
    <field:angular_calibration type="NX_FLOAT" optional="true" />
    <field:flatfield_applied type="NX_BOOLEAN" optional="true"/>
    <field:flatfield type="NX_FLOAT" optional="true" />
    <field:flatfield_error type="NX_FLOAT" optional="true" />
    <field:pixel_mask_applied type="NX_BOOLEAN" optional="true" />
    <field:pixel_mask type="NX_INT" recommended />
    <field:countrate_correction_applied type="NX_BOOLEAN" optional="true" />
    <field:bit_depth_readout type="NX_INT" recommended="true" />
    <field:detector_readout_time type="NX_FLOAT" units="NX_TIME" optional="true" />
    <field:frame_time type="NX_FLOAT" units="NX_TIME" optional="true" />
    <field:gain_setting type="NX_CHAR" optional="true" />
    <field:saturation_value type="NX_INT" optional="true" />
    <field:underload_value type="NX_INT" optional="true" />
    <field:sensor_material type="NX_CHAR" required />
    <field:sensor_thickness type="NX_FLOAT" units="NX_LENGTH" required />
    <field:threshold_energy type="NX_FLOAT" units="NX_ENERGY" optional="true" />
    <field:type optional />
</group:NXdetector>
<group:NXbeam required>
    <field:incident_wavelength type="NX_FLOAT" units="NX_WAVELENGTH" required />
    <field:incident_wavelength_weight type="NX_FLOAT" optional="true" />
    <field:incident_wavelength_spread type="NX_FLOAT" units="NX_WAVELENGTH" optional />
    <group name="incident_wavelength_spectrum" type="NXdata" optional="true" />
    <field:flux type="NX_FLOAT" units="NX_FLUX" optional>
    <field:"total_flux" type="NX_FLOAT" units="NX_FREQUENCY" required />
    <field:incident_beam_size type="NX_FLOAT" units="NX_LENGTH"  recommended="true" />
    <field:profile type="NX_CHAR" recommended="true" />
    <field:incident_polarisation_stokes recommended="true" />
```

```
            </group:NXbeam>
        </group:NXinstrument>

        <group:NXsource>
            <field:name required >
                <attribute:short_name" optional="true" />
            </field:name>
        </group:NXsource>

    </group:NXentry>
</definition>
```

2. NXmx Full Layout

This is a snapshot of the HDRMX NXmx application definition as it is being proposed to the NeXus International Advisory Committee (NIAC) for adoption by NIAC. That adoption process and discussions in the community are likely to result in additions to the Gold Standard as well as changes. The latest version prior to formal adoption is available from `http://github.com/HDRMX/definitions`. The HDRMX version will be updated as needed to reflect changes during and after adoption.

Status:

application definition, extends NXobject

Description:

functional application definition for macromolecular crystallography

Symbols:

These symbols will be used below to coordinate datasets of the same shape (configuration of array dimensions). Most MX x-ray detectors will produce two-dimensional images. Some will produce three-dimensional images, using one of the indices to select a detector module.

dataRank: rank of the `data` field

np: number of scan points

i: number of detector pixels in the slowest direction

j: number of detector pixels in the second slowest direction

k: number of detector pixels in the third slowest direction

m: number of channels in the incident beam spectrum, if known

Groups cited: NXattenuator, NXbeam, NXcollection, NXdata, NXdetector_group, NXdetector_module, NXdetector, NXentry, NXgeometry, NXinstrument, NXnote, NXsample, NXsource, NXtransformations

Structure:

ENTRY: (required) NXentry

Note, it is recommended that file_name and file_time be included as attributes at the root of a file that includes NXmx. See NXroot.

title: (optional) NX_CHAR

start_time: (required) NX_DATE_TIME

ISO 8601 time/date of the first data point collected in UTC, using the Z suffix to avoid confusion with local time. Note that the time zone of the beamline should be provided in NXentry/NXinstrument/time_zone.

end_time: (optional) NX_DATE_TIME

ISO 8601 time/date of the last data point collected in UTC, using the Z suffix to avoid confusion with local time. Note that the time zone of the beamline should be provided in NXentry/NXinstrument/time_zone. This field should only be filled when the value is accurately observed. If the data collection aborts or otherwise prevents accurate recording of the end_time, this field should be omitted.

end_time_estimated: (required) NX_DATE_TIME

ISO 8601 time/date of the last data point collected in UTC, using the Z suffix to avoid confusion with local time. Note that the time zone of the beamline should be provided in NXentry/NXinstrument/time_zone. This field may be filled with an estimated value before an observed value is available.

definition: (required) NX_CHAR

NeXus NXDL schema to which this file conforms

Obligatory value: `NXmx`

DATA: (required) NXdata

data[np, i, j, k]: (recommended) NX_NUMBER

For a 2-dimensional detector, the rank of the data array will be 3. For a 3-dimensional detector, the rank of the data array will be 4. This allows for the presentation of the frame number as the first index.

SAMPLE: (required) NXsample

name: (required) NX_CHAR Descriptive name of sample

depends_on: (required) NX_CHAR

This is a requirement for any scan experiment.

The axis on which the sample position depends may be stored anywhere but normally is stored in the NXtransformations group within the NXsample group.

If there is no goniometer, *e.g.* with a jet or other sample delivery system for which the positioning of each sample is not well-determined, depends_on should be set to "."

temperature: (optional) NX_CHAR {units=NX_TEMPERATURE}

TRANSFORMATIONS: (recommended) NXtransformations

This is the recommended location for sample goniometer and other related axes.

This is required for any scan experiment. The reason it is optional is primarily to accommodate XFEL single shot exposures.

Use of the depends_on field and the NXtransformations group is strongly recommended. As noted above this should be an absolute requirement to provide for any scan experiment.

The reason it is optional is mainly to accommodate XFEL single shot exposures.

INSTRUMENT: (required) NXinstrument

name: (required) NX_CHAR

Name of instrument. Consistency with the controlled vocabulary beamline naming in `http://mmcif.wwpdb.org/dictionaries/mmcif_pdbx_v50.dic/Items/_diffrn_source.pdbx_synchrotron_beamline.html` and `http://mmcif.wwpdb.org/dictionaries/mmcif_pdbx_v50.dic/Items/_diffrn_source.type.html` is highly recommended.

@short_name: (required) NX_CHAR

short name for instrument, perhaps the acronym.

time_zone: (recommended) NX_DATE_TIME

ISO 8601 time_zone offset from UTC

ATTENUATOR: (optional) NXattenuator

attenuator_transmission: (optional) NX_NUMBER

{units=NX_UNITLESS}

DETECTOR_GROUP: (recommended) NXdetector_group

Optional logical grouping of detector elements.

Each detector element is represented as an NXdetector group with its own detector data array. Each detector data array may be further decomposed into array sections by use of NXdetector_module groups. The names are given in the group_names field.

The groups are defined hierarchically, with names given in the group_names field, unique identifying indices given in the group_index field, and the level in the hierarchy given in the group_parent field. For example if an x-ray detector, DET, consists of four elements in a rectangular array:

```
DTL    DTR
DLL    DLR
```

we could have:

```
group_names:
  ["DET", "DTL", "DTR", "DLL", "DLR"]
group_index: [1, 2, 3, 4, 5]
group_parent:  [-1, 1, 1, 1, 1]
```

group_names: (required) NX_CHAR

An array of the names of the detector elements or hierarchical groupings of detector elements.

Specified in the base classes as a comma-separated list of names, but new code should use an array of names as quoted strings.

group_index[i]: (required) NX_INT

An array of unique indices for detector elements or groupings of detector elements.

Each element is a unique ID for the corresponding group named in the group_names field . The IDs are positive integers starting with 1.

group_parent[group_index]: (required) NX_INT

An array of the hierarchical levels of the parents of detector elements or groupings of detector elements.

A top-level element or grouping has parent level -1.

DETECTOR: (required) NXdetector

Normally the detector group will have the name `detector`. However, in the case of multiple detector elements, each element needs a uniquely named NXdetector group.

depends_on: (required) NX_CHAR

NeXus path to the detector positioner axis that most directly supports the detector.

data[np, i, j, k]: (recommended) NX_NUMBER

For a 2-dimensional detector, the rank of the data array will be

3. For a 3-dimensional detector, the rank of the data array will be 4. This allows for the presentation of the frame number as the first index.

description: (recommended) NX_CHAR

name/manufacturer/model/*etc.* information

time_per_channel: (optional) NX_NUMBER

{units=NX_TIME}

For a time-of-flight detector this is the scaling factor to convert from the numeric value reported to the flight time for a given measurement.

distance: (recommended) NX_FLOAT

{units=NX_LENGTH}

Distance from the sample to the beam center. Normally this value is for guidance only; the proper geometry can be found following the depends_on axis chain. But in appropriate cases where the dectector distance to the sample is observable independently of the axis chain, this may take precedence over the axis chain calculation.

distance_derived: (recommended) NX_BOOLEAN

{units=NX_LENGTH}

Boolean to indicate if the beam center is a derived, rather than a primary, observation. If distance_derived is true or is not specified, the distance is assumed to be derived from detector axis specifications.

dead_time: (optional) NX_FLOAT {units=NX_TIME}

Detector dead time

count_time: (recommended) NX_NUMBER

{units=NX_TIME}

Elapsed actual counting time.

beam_center_derived: (optional) NX_BOOLEAN

{units=NX_LENGTH}

Boolean to indicate if the distance is a derived, rather than a primary, observation. If not provided, the value of beam_center_derived is assumed to be true

beam_center_x: (recommended) NX_FLOAT

{units=NX_LENGTH}

This is the x coordinate in units of length of the position where the direct beam would hit the detector. Its location can be outside the actual detector. The length can be in physical units or pixels as documented by the units attribute. Normally, this should be derived from the axis chain, but the direct specification may take precedence if it is not a derived quantity.

beam_center_y: (recommended) NX_FLOAT

{units=NX_LENGTH}

This is the y coordinate in units of length of the position where the direct beam would hit the detector. Its location can be outside the actual detector. The length can be in physical units or pixels as documented by the units attribute. Normally, this should be derived from the axis chain, but the direct specification may take precedence if it is not a derived quantity.

angular_calibration_applied: (optional) NX_BOOLEAN

True when the angular calibration has been applied in the elec-

tronics, false otherwise.

angular_calibration[i, j, k]: (optional) NX_FLOAT

Angular calibration data.

flatfield_applied: (optional) NX_BOOLEAN

True when the flat field correction has been applied in the electronics, false otherwise.

flatfield[i, j, k]: (optional) NX_FLOAT

Flat field correction data. If provided, it is recommended that it be compressed.

flatfield_error[i, j, k]: (optional) NX_FLOAT

Errors in the flat field correction data. If provided, it is recommended that it be compressed.

pixel_mask_applied: (optional) NX_BOOLEAN

True when the pixel mask correction has been applied in the electronics, false otherwise.

pixel_mask[i, j]: (recommended) NX_INT

The 32-bit pixel mask for the detector. This can be either one mask for the whole dataset (i.e. an array with indices i, j) or each frame can have its own mask (in which case it would be an array with indices np, i, j). It contains a bit field for each pixel to signal dead, blind, high or otherwise unwanted or undesirable pixels. They have the following meaning:

- bit 0: gap (pixel with no sensor)
- bit 1: dead
- bit 2: under-responding
- bit 3: over-responding

- bit 4: noisy
- bit 5: -undefined-
- bit 6: pixel is part of a cluster of problematic pixels

(bit set in addition to others)

- bit 7: -undefined-
- bit 8: user-defined mask (e.g. around beamstop)
- bits 9 - 30: -undefined-
- bit 31: virtual pixel (corner pixel with interpolated value)

Normally data analysis software would not take pixels into account when a bit in (mask & 0x0000FFFF) is set. A tag bit in the upper two bytes indicates special pixel properties that normally would not be a sole reason to reject the intensity value unless lower bits are set.

If the full bit depth is not required, providing a mask with fewer bits is permissible.

If needed, additional pixel masks can be specified by including additional entries named pixel_mask_N, where N is an integer. For example, a general bad pixel mask could be specified in pixel_mask to indicate noisy and dead pixels, and an additional pixel mask from experiment-specific shadowing could be specified in pixel_mask_2. The cumulative mask is the bitwise OR of pixel_mask and any pixel_mask_N entries.

If provided, it is recommended that it be compressed.

countrate_correction_applied: (optional) NX_BOOLEAN

True when a count-rate correction has already been applied to the data recorded here, false otherwise.

bit_depth_readout: (recommended) NX_INT

The number of bits per pixel recorded by the electronics.

detector_readout_time: (optional) NX_FLOAT

{units=NX_TIME}

Time it takes to read the detector. This is important to know for time-resolved experiments.

frame_time: (optional) NX_FLOAT

{units=NX_TIME}

This is the time for each frame. This is exposure_time + readout_time.

gain_setting: (optional) NX_CHAR

The gain setting of the detector. This influences background.

saturation_value: (optional) NX_INT

The value at which the detector goes into saturation. Data above this value is known to be invalid.

For example, given a saturation_value and an underload_value, the valid pixels are those less than or equal to the saturation_value and greater than or equal to the underload_value.

underload_value: (optional) NX_INT

The lowest value at which pixels for this detector would be reasonably measured.

For example, given a saturation_value and an underload_value, the valid pixels are those less than or equal to the saturation_value and greater than or equal to the underload_value.

sensor_material: (required) NX_CHAR

At times, radiation is not directly sensed by the detector. Rather,

the detector might sense the output from some converter like a scintillator. This is the name of this converter material.

sensor_thickness: (required) NX_FLOAT

{units=NX_LENGTH}

At times, radiation is not directly sensed by the detector. Rather, the detector might sense the output from some converter like a scintillator. This is the thickness of this converter material.

threshold_energy: (optional) NX_FLOAT

{units=NX_ENERGY}

Single photon counter detectors can be adjusted for a certain energy range in which they work optimally. This is the minimum energy setting for this collection. If the detector supports multiple thresholds, this is an array.

type: (optional) NX_CHAR

Description of type such as scintillator, ccd, pixel, image plate, CMOS, ...

TRANSFORMATIONS: (recommended) NXtransformations

Location for axes (transformations) related to the detector.

COLLECTION: (optional) NXcollection

Suggested container for detailed non-standard detector information like corrections applied automatically or performance settings.

DETECTOR_MODULE: (required) NXdetector_module

Many detectors consist of multiple smaller modules that operate together and store their data in a common dataset. To allow consistent parsing of the experimental geometry, this application

definition requires all detectors to define a detector module, even if there is only one.

This group specifies the hyperslab of data in the data array associated with the detector that contains the data for this module. If the module is associated with a full data array, rather than with a hyperslab within a larger array, then a single module should be defined, spanning the entire array.

data_origin: (required) NX_INT

A 2-dimensional or 3-dimensional field which gives the indices of the origin of the hyperslab of data for this module in the main area detector image in the parent NXdetector module.

The data_origin is 0-based.

The frame number dimension (np) is omitted. Thus the data_origin field for a 2-dimensional dataset with indices (np, i, j) will be an array with indices (i, j), and for a 3-dimensional dataset with indices (np, i, j, k) it will be an array with indices (i, j, k).

The order of indices (i, j) or (i, j, k) is slow to fast.

data_size: (required) NX_INT

Two or three values for the size of the module in pixels in each direction. Dimensionality and order of indices is the same as for data_origin.

data_stride: (optional) NX_INT

Two or three values for the stride of the module in pixels in each direction. By default the stride is [1,1] or [1,1,1], and this is the most likely case. This optional field is included

for completeness.

module_offset: (optional) NX_NUMBER

{units=NX_LENGTH}

Offset of the module in regards to the origin of the detector in an arbitrary direction.

@transformation_type: (required) NX_CHAR

Obligatory value: `translation`

@vector: (required) NX_NUMBER

@offset: (required) NX_NUMBER

@depends_on: (required) NX_CHAR

fast_pixel_direction: (required) NX_NUMBER

{units=NX_LENGTH}

Array of values along the fast-varying pixel direction. The direction itself is given by the vector attribute.

@transformation_type: (required) NX_CHAR

Obligatory value: `translation`

@vector: (required) NX_NUMBER

@offset: (required) NX_NUMBER

@depends_on: (required) NX_CHAR

slow_pixel_direction: (required) NX_NUMBER

{units=NX_LENGTH}

Array of values along the slow-varying pixel direction. The direction itself is given by the vector attribute.

@transformation_type: (required) NX_CHAR

Obligatory value: `translation`

@vector: (required) NX_NUMBER

@offset: (required) NX_NUMBER

@depends_on: (required) NX_CHAR

BEAM: (required) NXbeam

incident_wavelength: (required) NX_FLOAT

{units=NX_WAVELENGTH}

In the case of a monochromatic beam this is the scalar wavelength.

Several other use cases are permitted, depending on the presence or absence of other incident_wavelength_X fields.

In the case of a polychromatic beam this is an array of length **m** of wavelengths, with the relative weights given in incident_wavelength_weight.

In the case of a monochromatic beam that varies shot-to-shot, this is an array of wavelengths, one for each recorded shot. Here, incident_wavelength_weight and incident_wavelength_spread are not set.

In the case of a polychromatic beam that varies shot-to-shot, this is an array of length **m** with the relative weights specified in incident_wavelength_weight as a 2D array.

In the case of a polychromatic beam that varies shot-to-shot and where the channels also vary, this is a 2D array of dimensions **np** by **m** (slow to fast) with the relative weights specified in incident_wavelength_weight as a 2D array.

incident_wavelength_weight: (optional) NX_FLOAT

In the case of a polychromatic beam this is an array of length **m** of the relative weights of the corresponding wavelengths specified in incident_wavelength.

In the case of a polychromatic beam that varies shot-to-shot, this is a 2D array of dimensions **np** by **m** (slow to fast) of the relative weights of the corresponding wavelengths specified in incident_wavelength.

incident_wavelength_spread: (optional) NX_FLOAT {units=NX_WAVELENGTH}

The wavelength spread full width at half max (FWHM) for the corresponding wavelength(s) specified in incident_wavelength.

In the case of shot-to-shot variation in the wavelength spread, this is a 2D array of dimensions **np** by **m** (slow to fast) of the spreads of the corresponding wavelengths specified in incident_wavelength.

flux: (optional) NX_FLOAT {units=NX_FLUX}

Flux density incident on beam plane area in photons per second per unit area.

In the case of a beam that varies in flux shot-to-shot, this is an array of values, one for each recorded shot.

total_flux: (required) NX_FLOAT {units=NX_FREQUENCY}

Flux incident on beam plane in photons per second.

In the case of a beam that varies in total flux shot-to-shot, this is an array of values, one for each recorded shot.

incident_beam_size[2]: (recommended) NX_FLOAT {units=NX_LENGTH}

Two-element array of full width at half max (FWHM) if Gaussian or Airy function, diameters if top hat, or widths if rectangular, of the beam in the order x, y.

profile: (recommended) NX_CHAR

The beam profile: Gaussian, Airy function, top-hat, or rectangular. The profile is given in the plane of incidence of the beam on the sample.

Any of these values: `Gaussian`, `Airy`, `top-hat`, `rectangular`.

incident_polarisation_stokes[np, 4]: (recommended) NX_CHAR

incident_wavelength_spectrum: (optional) NXdata

SOURCE: (required) NXsource

The neutron or x-ray storage ring/facility. Note, the NXsource base class has many more fields available, but at present we only require the name.

name: (required) NX_CHAR

Name of source. Consistency with the naming in `http://mmcif.wwpdb.org/dictionaries/mmcif_pdbx_v50.dic/Items/_diffrn_source.pdbx_synchrotron_site.html` controlled vocabulary is highly recommended.

@short_name: (optional) NX_CHAR

short name for source, perhaps the acronym.
